# Cell-wall-degrading enzymes produced in vitro and in vivo by *Rhizoctonia solani*, the causative fungus of peanut sheath blight

**DOI:** 10.7717/peerj.5580

**Published:** 2018-09-05

**Authors:** Cai Yun Xue, Ru Jun Zhou, Yuan Jie Li, Di Xiao, Jun Fan Fu

**Affiliations:** Department of Plant Protection, Shenyang Agriculture University, Shenyang, Liaoning, China

**Keywords:** Peanut, Peanut sheath blight disease, *Rhizoctonia solani*, Cell-wall-degrading enzymes, IEF

## Abstract

*Rhizoctonia solani* causes the disease peanut sheath blight, involving symptoms of maceration and necrosis of infected tissue, mainly caused by cell-wall-degrading enzymes (CWDEs). This study investigated the production of CWDEs including polygalacturonase (PG), polymethyl-galacturonase (PMG), cellulase (Cx) and β-glucosidase by *R. solani* in vitro (in liquid culture) and in vivo (in peanut plants). Significant PG, PMG, Cx and β-glucosidase activities were detected in infected tissues including stalk and leaves of Baisha and Silihong peanut cultivars. Extracts of healthy tissue showed little or no such activities. In shaken liquid cultures of *R. solani* in medium containing pectin or pectin plus carboxymethyl cellulose (CMC) as the carbon source(s), PG and PMG were notably active. Significant Cx activity was detected in cultures with CMC or pectin plus CMC as the carbon source(s). However, only a very low level of β-glucosidase activity was observed in cultures with any of the tested carbon sources. An increase of pH was recorded in decayed peanut tissues and liquid culture filtrates; the filtrate pH and fungal growth positively correlated. The fungal growth and/or pH were important factors for the production of PG, PMG and Cx in culture with pectin plus CMC as the carbon source. A single active PG isozyme with isoelectric point around 9.2 was detected in culture filtrates and in infected peanut tissues by the method of isoelectric focusing electrophoresis. The crude enzymes extracted from liquid culture of *R. solani* induced decay of healthy peanut leaves.

## Introduction

Most plant fungal pathogens produce a wide range of cell-wall-degrading enzymes (CWDEs) (Zhang et al., 1999; Lalaoui et al., 2000; Gao et al., 2000; Brien & Zamani, 2003; Yang et al., 2012b; Li et al., 2012; Zhao et al., 2014; Zhang, Bruton & Biles, 2014; Amit et al., 2014). As determinants of the fungal pathogenicity complex, CWDEs may be important to fungi not only for penetration and hyphal branching inside the plant tissue, but also for releasing nutrients from the wall polysaccharides that are necessary for fungal growth (De Lorenzo et al., 1997). Correlation has been confirmed between the levels of CWDE activity and the degree of pathogenesis (Jia et al., 2009; Zhang, Bruton & Biles, 2014; Zhou et al., 2016; Gawade et al., 2017). It has been demonstrated histochemically and histopathologically that the disintegration and change of plant cell walls is due to CWDEs (Weinhold & Motta, 1973; Fehrmann & Mendgen, 1975; Wanjiru, Kang & Buchenauer, 2002; Kang et al., 2007).

Cell-wall-degrading enzymes, especially pectolytic enzymes, play a decisive role in the process of fungal infection of plants by degrading pectins, which provides a carbon source for the pathogen as well as exposing cell wall components to other enzymes such as cellulases (Cxs) and hemicellulases for further cell wall breakdown (Wiethölter et al., 2003; Jia et al., 2009). Polygalacturonase (PG) and polymethyl-galacturonase (PMG), respectively, break down pectate and pectin, by hydrolysis; they cleave the α-(1,4)-glycosidic bonds in the d-galacturonic acid moieties of the pectic substances with the introduction of water across the oxygen bridge. It has been demonstrated that the ability of pathogenic fungi to produce large quantities of PG in culture and in inoculated tissue is correlated with their virulence (Lalaoui et al., 2000; Douaiher et al., 2007b; Zhang, Bruton & Biles, 2014; Zhang et al., 1999; Gawade et al., 2017). PMG also shows high activity in culture and in host tissue inoculated with many fungal pathogens and has been determined to be a virulence factor (Gao et al., 2000; Li et al., 2003, 2012; Chen et al., 2006; Dong et al., 2010; Yang et al., 2012b; Zhou et al., 2016).

The degradation of the plant cell wall component cellulose in the process of infection is due to the activity of Cxs produced by the pathogen. Cxs are a group of enzymes including endoglucanase (Cx; EC 3.2.1.4), cellobiohydrolase (C_1_; EC 3.2.1.91) and β-glucosidase (EC 3.2.1.21) (Sipos et al., 2010; Zhang, Bruton & Biles, 2014). Cx degrades cellulose to cellobiose and is related to the virulence of pathogens (Ikotun, 1984; Lalaoui et al., 2000; Yang et al., 2012b; Zhao et al., 2014; Zhang, Bruton & Biles, 2014; Zhou et al., 2016). Many plant fungal pathogens have been reported to produce Cx in cultivation in infected host tissue, or both (Ikotun, 1984; Lalaoui et al., 2000; Gao et al., 2000; Li et al., 2003; Chen et al., 2006; Douaiher et al., 2007a; Dong et al., 2010; Yang et al., 2012b; Zhang, Bruton & Biles, 2014; Zhao et al., 2014; Zhou et al., 2016). The activity of Cx produced by *Didymella bryoniae* in cultures and in decayed tissue positively correlated with disease severity (Zhang, Bruton & Biles, 2014; Zhou et al., 2016). β-glucosidase acts on cellobiose and cleaves it into glucose monomers (Sipos et al., 2010); this activity has been observed in culture and in diseased tissue inoculated with pathogens such as *Thanatephorus cucumeris* (Jayasinghe, Wijayaratne & Fernando, 2004; Zhao et al., 2014), *Phaeosphaeria nodorum* (Lalaoui et al., 2000), *Colletotrichum acutatum* (Fernando, Jayasinghe & Wijesundera, 2001), *Gaeumannomyces graminis* (Dori, Solel & Barash, 1995) and *Fusarium sulphureum* (Yang et al., 2012b).

Cell-wall-degrading enzymes have been extensively studied by gene disruption, in replacement experiments and as recombinant proteins. Several PG genes have been obtained from plant fungal pathogens and it was determined that most PG genes play an important role in pathogenicity (Shieh et al., 1997; Di Pietro & Roncero, 1998; Oeser et al., 2002; Yang et al., 2012a; Chen et al., 2014, 2017a, 2017b). Cx gene fragments were amplified from *F. oxysporum* (Sheppard et al., 1994). Afterward, Cx genes *egl1*, *egl2* and *PlEGL1* from the pathogens *Macrophomina phaseolina* and *Pyrenochaeta lycopersici* were cloned and their function was determined (Wang & Jones, 1995a, 1995b; Valente, Infantino & Aragona, 2011). β-glucosidase genes have been cloned from *Sclerotinia sclerotiorum, Phaeosphaeria nodorum and Phaeosphaeria avenaria* f.sp. *triticea* (Waksman, 1989; Reszka et al., 2005). High activities of PMG were also previously detected in cultivation or in infected host tissue. However, the function of PMG genes from plant fungal pathogens has not been investigated extensively.

Most plant-pathogenic fungi secrete multiple isozymes of CWDEs that differ in isoelectric point and molecular weight (Dori, Solel & Barash, 1995; Martel, Létoublon & Fěvre, 1996; Le Cam et al., 1997; Zhang et al., 1999; Cabanne & Donèche, 2002; Schnitzhofer et al., 2007; Dong et al., 2010). Meanwhile, the pathogens may produce different types of isozymes in culture and in infected tissue (Zhang et al., 1999), in different host tissues, and even during different periods of the same infection (Zhang et al., 1999; Jurick et al., 2010). The multiplicity of isozymes may be associated with the virulence of the pathogen, and give flexibility to the pathogen. Each isozyme has its own unique properties that contribute to the pathogenicity to the host (Dong et al., 2010). However, little study has been concerned with isozymes in different infected parts of plant tissue.

*Rhizoctonia solani* Kuhn (the teleomorph of *T. cucumeris*) is a cosmopolitan pathogen in soils that can destroy a wide host range of plants including rice, corn, potato, tomato, *Mentha*, bean, soybean, lucerne and lettuce, in both tropical and temperate parts of the world (Taheri, Gnanamanickam & Höfte, 2007; Li, Wu & Yan, 1998; Chen et al., 2016; Bartz et al., 2010; Nitzan et al., 2012; Spedaletti et al., 2016; Fenille, De Souza & Kuramae, 2002; Anderson et al., 2004; Grosch, Schneider & Kofoet, 2004). Peanut sheath blight disease caused by *R. solani* has been reported in Liaoning Province, China. The symptoms are water-soaked lesions, necrosis of leaves, and web-like mycelia on plants, followed by the development of sclerotia on tissue surfaces. The disease in peanut occurs severely between mid-July and mid-August, during high humidity and high temperature (19–35 °C) season (Fu et al., 2014). The characteristic symptoms of peanut sheath blight including necrosis and maceration of the infected tissue indicate the possible involvement of CWDEs in the process of disease establishment and development (Jayasinghe, Wijayaratne & Fernando, 2004). The CWDEs associated with *R. solani* pathogenesis have been investigated in several crops, such as sugar beet (El-Abyad, Abu-Taleb & Abdel-Mawgoud, 1997), rubber (Jayasinghe, Wijayaratne & Fernando, 2004) and rice (Chen et al., 2006). However, to our knowledge, no literature is available on the involvement of CWDEs in the development of peanut sheath blight.

The present study was undertaken to: (1) investigate and quantify the activity of CWDEs of *R. solani* produced in liquid culture and during infection of peanut tissue; (2) to investigate the involvement crude enzyme extracts (primarily focused on CWDEs) in disease development in peanut leaves; and (3) to identify PG isoenzymes produced by *R. solani*.

## Material and Methods

### Fungal isolate

*R. solani* isolate CLL-01 used in this study was collected from naturally infected peanut tissue in a field in Wangjia Town, Liaoyang City, Liaoning Province, China. The isolate was cultured on potato dextrose agar (PDA) at 25 °C for 36 h and identified as AG1-IA by molecular evidence (NCBI Accession number: MH395844, with 99% identity to *R. solani* AG1-IA isolated from *Salvia miltiorrhiza*; also available in Supplemental Dataset S1) (Yi et al., 2016).

### Enzyme production and growth in liquid culture

A modified Richard’s liquid medium (Zhang, Bruton & Biles, 2014) containing KNO_3_ (10 g l^−1^), KH_2_PO_4_ (5 g l^−1^), MgSO_4_·7H_2_O (2.5 g l^−1^) and FeCl_3_·6H_2_O (0.02 g l^−1^) was used. Carbon sources used in the liquid medium were: 1% (w/v) orange pectin, 0.5% (w/v) orange pectin + 0.5% (w/v) carboxymethyl cellulose (CMC), or 1% (w/v) CMC. The initial pH of autoclaved liquid medium containing pectin, pectin plus CMC, or CMC was measured before inoculation. Liquid medium (100 ml) was dispensed into 250 ml flasks, which were inoculated with two 0.5 cm. PDA plugs obtained from the periphery of 36-h-old cultures of the isolate growing at 25 °C. The flasks were placed on an orbital shaker (100 rpm) at 25 °C. Every second day, from day 2 until day 16, the contents were filtered through Miracloth™ (Calbiochem, EMD Millipore Corp., Billerica, MA, USA). The filtrates were centrifuged at 16,000×*g* for 20 min and stored at −20 °C until use for detection of enzyme activity. The pH of the filtrates was determined using a pH electrode (PB-10; Sartorius, Gottingen, Germany) soon after harvest. Fungal mycelia were removed using the Miracloth and then both the mycelia and Miracloth were dried to constant weight at 80 °C and weighed. The Miracloth was pre-dried and weighed for fungal mass calculation.

### Plant material and inoculation

Two peanut cultivars, Baisha (Bs) and Silihong (Slh), were used for the following examination. The peanut seeds were surface-sterilized with 70% ethanol for 10 s followed by 1.4% NaOCl for 20 min, then extensively washed with sterile water. The seeds were germinated in sterile conditions at 25 °C. Three germinant peanut seeds were placed in each pot (10 cm diameter, seven cm depth) containing a sterile potting mix of soil and peat moss (4:1). The peanut plants were grown in a greenhouse at 27 ± 1 °C, humidity 60∼99%, day 14∼15 h and night 9∼10 h, at light intensity of 8.6∼61.0 klx.

Inoculum was produced for in vivo experiments in peanut using the following procedures. Isolate CLL-01 was grown on PDA at 25 °C for 36 h. Two 0.5 cm diameter mycelial plugs obtained from the periphery of the culture were added to a 250 ml flask containing 100 g sterile corn niblets which had been boiled for 30 min and dipped in water overnight before autoclaving. The corn kernel inocula was incubated at 25 °C for 5 days. The flasks were shaken once per day to ensure the grains were evenly and fully incubated. Five cultured kernels were placed approximately one cm deep in the soil around the base of each 5-week-old peanut plant. Each pot of three plants was placed in a clear plastic bag as needed to maintain high humidity. The infected stalk and leaf tissues were collected 10 days post-inoculation (dpi) and stored at −20 °C until enzyme extraction. The controls were inoculated with sterile corn kernels. The pH values of exudate of the control and infected peanut samples were examined using precise pH indicator paper.

### Extraction of enzymes from infected tissue

The extraction of PG, PMG, Cx and β-glucosidase from decayed or healthy peanut plant tissue was conducted according to procedures described previously (Zhang et al., 1999) with some modifications. Decayed tissue (one g) was placed in five ml of 0.1M Na-acetate buffer (pH 5.0) containing 1M NaCl and one mM EDTA in a precooled mortar, on ice. The mixture was ground with silica sand for 2 min. The slurry was filtered through Miracloth, followed by centrifugation of filtrates at 16,000×*g* for 20 min, and then the supernatants were collected and stored at −20 °C until further processing.

### PG and PMG activity assays

Polygalacturonase activity was assayed by the 3,5-dinitrosalicylic acid (DNS) method, as described by Cao, Jiang & Zhao (2007). The substrate was 1% polygalacturonic acid in 50 mM Na-acetate buffer (pH 5.5). The reaction mixture consisted of 0.5 ml sample, 1.0 ml Na-acetate buffer (50 mM, pH 5.5) and 0.5 ml substrate, and it was incubated for 60 min at 37 °C. The reaction was terminated by adding 1.5 ml DNS and boiling for 5 min, and the absorbance was measured at 540 nm using a Spectrophotometer (Multiskan GO; ThermoFisher Scientific, Boston, USA). Boiled enzyme was used as a control. Galacturonic acid at various concentrations was used to establish a standard curve. Activity of PMG was measured using the same procedure as that used for assaying PG activity, except that the substrate was 1% pectin in 50 mM Na-acetate buffer (pH 5.5).

### Cx and β-glucosidase activity assays

Cellulase activity was assayed by the DNS method as described by Cao, Jiang & Zhao (2007). The substrate was 1% CMC in 50 mM citric acid-sodium citrate buffer (pH 5.0). The reaction mixture consisted of 0.5 ml sample and 1.5 ml substrate, and it was incubated for 60 min at 37 °C. The reaction was terminated by adding 1.5 ml DNS and boiling for 5 min, and the absorbance was measured at 540 nm using a Spectrophotometer. Boiled enzyme was used as a control. Glucose at various concentrations was used to establish a standard curve. Activity of β-glucosidase was measured using the same procedure as that used for assaying Cx activity, except that the substrate was 1% salicin in 50 mM citric acid-sodium citrate buffer (pH 4.5).

In this study, one unit of enzyme activity was defined as one μmol of reducing groups released by the enzyme per min at 37 °C. All enzyme activities of culture extracts are expressed in units ml^−1^, while those in infected tissue extracts are expressed in units mg^−1^ protein.

### Protein assay

Protein concentrations were estimated by the method of Bradford (Cao, Jiang & Zhao, 2007). Bovine serum albumin was used to establish a standard curve.

### Preparation of crude enzyme extracts

The culture filtrate from medium containing 0.5% (w/v) orange pectin plus 0.5% (w/v) CMC, collected 10 dpi as described above, was used for the preparation of crude enzymes as this material showed the greatest PG enzyme activity. Ammonium sulfate was added to the culture filtrate to 90% saturation and incubated at 4 °C for 24 h, followed by centrifugation at 16,000×*g* for 30 min at 4 °C. The precipitate was dissolved in distilled water and extensively dialyzed (MWCO 10,000 Da) over 24 h at 4 °C against distilled water (replaced every 8 h), and then freeze-dried. The obtained crude enzyme extracts were stored at −20 °C until inoculation and use in ultrathin layer polyacrylamide isoelectric focusing (IEF) electrophoresis.

### Effects of crude enzyme extracts on peanut plant tissue

We examined the effect of the above crude enzymes extracted from culture filtrate on peanut plant tissue. The crude enzyme preparation from 10 dpi shaken culture with pectin plus CMC as the carbon source was inoculated onto peanut leaf (Bs cultivar). Two five μl drops of crude enzyme were placed on one side of the midrib of leaves and controls including heated crude enzyme extracts and distilled water were placed symmetrically on the other side. Incubation was in moist chambers at 27 °C, while the petiole was moistened with cotton wool and the bottom of the box contained water. Observations were made every 24 h for 7 days. To compare symptoms induced by crude enzyme extract and pathogens, a mycelial plug (five mm in diameter) obtained from 36-h-old cultures of the *R. solani* isolate CLL-01 growing on PDA at 25 °C was placed on the adaxial surface of the leaf at the main vein center with the mycelial side facing the leaf and incubated in a moist chamber in the same conditions. Sterile PDA plugs were used as controls.

### Isoelectric focusing electrophoresis of PG

Extract from 10 dpi peanut plant tissue (two g) was performed in 10 ml 0.1M Na-acetate buffer (pH 5.0) containing 1M NaCl and one mM EDTA, and then dialyzed and freeze-dried in the same manner as extracts from medium described in the section “crude enzyme extraction” but without ammonium sulfate precipitation. The crude enzymes from culture filtrate and infected tissue extract were subjected to thin-layer polyacrylamide gel IEF (Liuyi, DYCP-37B, Beijing, China) and evaluated for PG isoenzymes. The IEF used 0.4 mm thick polyacrylamide gels containing 4% (v/v) ampholytes (Bio-Lyte 3/10, 40%; BIO-RAD, Hercules, CA, USA) covering the pH range 3.5–10.0. The electrode strips were soaked with 1M phosphoric acid (anode) and 1M sodium hydroxide (cathode) and placed on opposing sides of the gel. The pre-run was carried out at 100–150 V for 30 min, followed the application of 20 μl protein samples to application strips placed directly on the gel. Heat-treated crude enzyme solution was used as a negative control. IEF protein standards (Isoelectric Focusing Calibration Kit, Broad pH 3–10; GE Healthcare, Amersham, UK) were diluted according to the manufacturer’s instructions before loading. After electrophoresis at 100–200 V for 1–1.5 h, the application strips were removed. Thereafter, electrophoresis was conducted at constant 580 V for about 3.5 h at the same temperature. Ultrathin overlay gels (0.9 mm) for PG isoenzyme detection were prepared as described by Ried & Collmer (1985) with the following modifications: the 10 g l^−1^ agarose gel contained one g l^−1^ polygalacturonic acid and 10 mM EDTA buffered at pH 5.0 with 50 mM Na-acetate. The polyacrylamide IEF gels overlaid with ultrathin agarose gels for PG detection were incubated at 100% humidity and 37 °C for 30–60 min. To visualize activity bands, the agarose overlay was stained for 10 min in 0.5 g l^−1^ ruthenium red solution. PG appeared as a white band visualized using a light box. The pI values of the PG isoenzymes were estimated from a regression equation of standard proteins vs. the distance migrated.

### Statistical evaluation

Statistical evaluation of the results obtained was performed using SPSS 20.0. Correlation analysis was performed between pH and mycelial dry weight, pH and enzyme production, and mycelial dry weight and enzyme production, respectively, for shaken-flask cultures.

## Results

### Production of cell-wall-degrading enzymes in infected tissue

**Figure 1:**
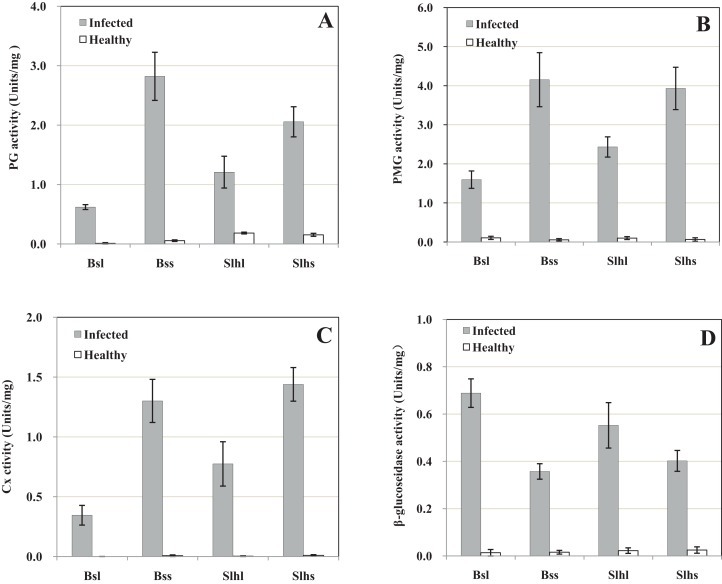
Activity of cell-wall-degrading enzymes including polygalacturonase (PG), polymethyl-galacturonase (PMG), cellulase (Cx) and β-glucosidase in peanut tissue infected by *R. solani* and healthy tissue. Healthy tissues were inoculated with sterile corn kernels. Samples were collected 10 days post-infection (dpi). (A) PG activity. (B) PMG activity. (C) Cx activity. (D) β-glucosidase activity. Bsl, Baisha cultivar leaf; Bss, Baisha cultivar stalk; Slhl, Silihong cultivar leaf; Slhs, Silihong cultivar stalk. Standard errors (vertical bars) were calculated from three replicates.

The pH of the exudate from healthy tissue including stalks and leaves of both Bs and Slh cultivars was 5.4, whereas from rotted tissue this increased to 6.7. The extracts of infected leaf and stalk of the two peanut cultivars showed notable PG activities, while healthy tissue showed only trace activity (Fig. 1A). The PG activities of the extract of infected leaf and stalk of Bs cultivar were, respectively, 56.7- and 51.0-fold those in healthy tissue; for Slh the activities were 6.6- and 13.4-fold those in healthy tissue, respectively. These results indicate that peanut tissue was alkalified in the infection process and high PG activity was induced by fungal infection.

The extracts of infected tissue showed high PMG activity, while healthy tissue showed very low level activity (Fig. 1B). The PMG activities of the extracts of infected leaf and stalk of Bs were 15.0- and 73.9-fold those in healthy tissue, respectively, and for Slh the activities were 24.2- and 70.4-fold those in healthy tissue. These results indicate that high PMG activity was induced by fungal infection.

Only traces of Cx activity were detected in the extract of healthy tissue, and no activity was detectable in extracts of leaf from the Bs cultivar. However, high Cx activity was detected in extracts of infected tissue, including leaf and stalk of both cultivars (Fig. 1C). The Cx activity of extract from infected Bs stalk was 188.6-fold that in healthy tissue, and the activities in infected Slh leaf and stalk were 302.6- and 145.7-fold those in healthy tissue, respectively. These results indicate that relatively high Cx activity was induced by fungal infection.

Very low β-glucosidase activity was detected in the extract of healthy tissue from the two cultivars. The β-glucosidase activity of the extract of infected tissue was higher (Fig. 1D): the β-glucosidase activities of the extract of infected leaf and stalk of Bs were, respectively, 50.7- and 22.9-fold those in healthy tissue, and for Slh the relative increases in activity were 24.6- and 23.2-fold. These results indicate that relatively high β-glucosidase activity was induced by fungal infection.

### Production of cell-wall-degrading enzymes in liquid media (in vitro culture)

**Figure 2:**
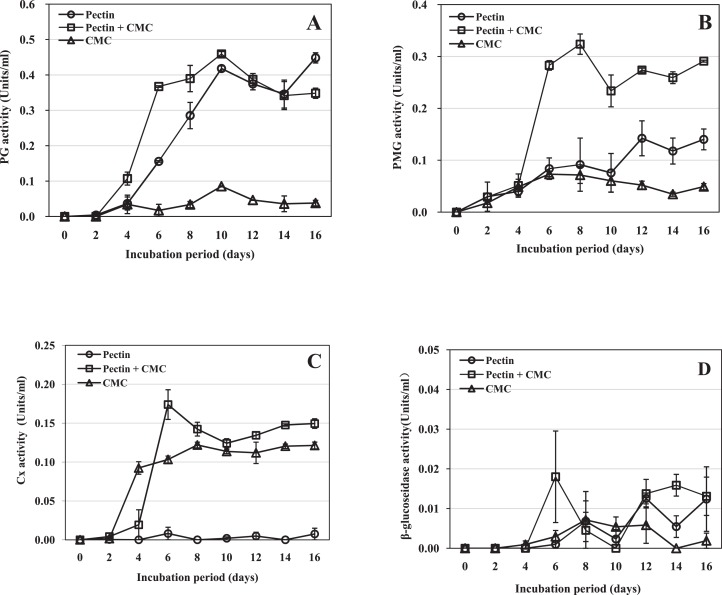
Activity of *R. solani* cell-wall-degrading enzymes including PG, PMG, Cx and β-glucosidase in liquid shaken cultures containing various carbon sources. Liquid medium with various carbon sources including 1% pectin, 0.5% pectin + 0.5% CMC or 1% CMC were used in the experiment, grown at 25 °C with shaking at 100 rpm. (A) PG activity. (B) PMG activity. (C) Cx activity. (D) β-glucosidase activity. Standard errors (vertical bars) were calculated from three replicates.

Polygalacturonase activity was first detected two dpi in the medium containing pectin as the sole carbon source, and four dpi in the medium containing CMC alone or pectin plus CMC as the carbon source(s) (Fig. 2A). In the three types of medium, the PG activity continued to increase to a peak on day 10, and then it tended to decrease until the end of the experiment on day 16. The highest amount of PG was produced in the medium containing pectin plus CMC, a little more than in medium with pectin alone, while the PG activity was low throughout the cultivation period in the CMC medium (Fig. 2A). On day 10 (the peak activity), PG activity in the medium containing pectin and pectin plus CMC was 4.90- and 5.38-fold that in the CMC-containing medium, respectively. The results indicated that PG activity was induced in the three media; the highest amount of PG activity was produced in the medium containing pectin plus CMC, followed by medium with pectin as the sole carbon source, and low activity was produced in CMC-containing medium.

Polymethyl-galacturonase activity was detected two dpi in all three types of medium (Fig. 2B). The activity in the medium containing pectin plus CMC reached its maximum on day 8 and remained high thereafter. On day 8, the activity was 3.5- and 4.5-fold that in the medium containing pectin or CMC as the carbon source, respectively. The activity in medium containing pectin as the sole carbon source peaked on day 12 and was 2.7-fold the activity in the medium containing CMC alone on that day, but was much lower than that in the medium containing pectin plus CMC. The activity in the medium containing CMC as the sole carbon source peaked on day 6 and then began to decrease; this medium resulted in the lowest PMG activity (Fig. 2B). The results indicated that PMG activity was induced in the three media. The highest activity was produced in medium containing pectin plus CMC, followed by medium with pectin as the sole carbon source, and then CMC-containing medium.

Cellulase activity was first detected two dpi in the medium containing CMC or pectin plus CMC, and six dpi in the medium containing pectin as the sole carbon source (Fig. 2C). In the medium containing pectin plus CMC, the activity increased rapidly to reach its maximum at day 6, when it was 21.4- and 1.7-fold the activity in the medium containing pectin or CMC as the carbon source, respectively; thereafter the activity remained high until the end of the experiment (day 16). In CMC-containing medium, the activity reached its maximum on day 8; the level of the activity was slightly lower than that in the medium containing pectin plus CMC through most of the cultivation process (Fig. 2C). In the medium containing pectin alone, only trace Cx activity was observed throughout the cultivation. The results indicated that Cx activity was induced in the three media; the highest activity was produced in the medium containing pectin plus CMC, followed by medium with CMC as the sole carbon source, and trace Cx activity was produced in pectin-containing medium.

β-glucosidase activity was low in all three types of medium. Activity was first detected at four dpi in the medium containing CMC alone; it peaked on day 8, followed by a decrease (Fig. 2D). β-glucosidase activity was first detected on day 6 in the other two media. The activity in the medium containing pectin plus CMC, or pectin alone, varied throughout the remainder of the experimental period. These results indicate that only trace β-glucosidase activity was induced in the three media.

### Fungal growth and culture filtrate pH in vitro

**Figure 3:**
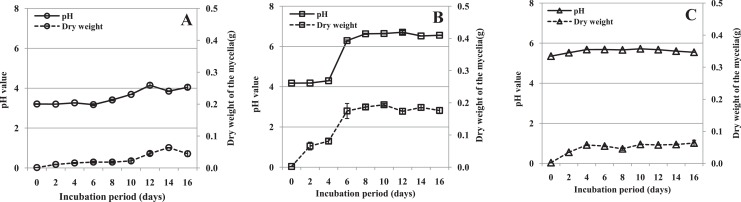
pH value of the culture filtrate and mycelial dry weight of *R. solani* in liquid shaken cultures containing various carbon sources. Liquid medium with various carbon sources including 1% pectin, 0.5% pectin + 0.5% CMC or 1% CMC were used in the experiment, grown at 25 °C with shaking at 100 rpm. (A) Pectin as the sole carbon source. (B) Pectin plus CMC as the carbon source. (C) CMC as the sole carbon source. Standard errors (vertical bars) were calculated from three replicates.

The mycelial dry weight and pH in the three media were determined after filtrates were obtained. The growth of mycelium was different in the three media. In medium containing pectin as the sole carbon source, the amount of mycelium remained low and roughly constant to 10 dpi, and then increased (Fig. 3A). In medium containing pectin and CMC, mycelium grew slowly between day 0 and 4, and rapidly from day 4 to 6, after which the growth rate levelled off. The overall growth rate was much higher than in the other two media (Fig. 3B). In the medium containing CMC as the sole carbon source, the mycelium increased a little between day 0 and 4 and then remained roughly constant throughout the experimental period (Fig. 3C). The results indicated that mycelium grew rapidly in medium containing pectin and CMC, but slowly in the other two media.

During growth, the pH of the culture medium increased (from different starting values) coincident with the increasing mycelial dry weight. The pH of the medium containing pectin plus CMC increased sharply after 4 days, and that of the other two media slightly accumulated through the experiment (Fig. 3). The correlations between pH value and mycelial dry weight were found to be significant in shaken cultures containing pectin (*r* = 0.837, *p* = 0.005), pectin plus CMC (*r* = 0.953, *p* = 0.000), and CMC (*r* = 0.803, *p* = 0.009). Moreover, we found that the production of PG, PMG and Cx appeared to have a similar pattern to the mycelial growth and pH in the culture containing pectin plus CMC (Figs. 2A–2C and 3B). Hence, the correlations between enzyme activity and pH of the culture, and enzyme activity and mycelial growth, were respectively analyzed. PG, PMG and Cx activity were found to correlate extremely significantly with pH and fungal growth in pectin plus CMC shaken cultures (Table 1), showing strong association between the activity of these enzymes and the culture pH, and between activity of the enzymes and fungal growth. PG and PMG activity in shaken cultures in medium containing pectin or CMC as the sole carbon source, Cx activity in CMC medium, and β-glucosidase in pectin medium showed lower correlations with pH and fungal growth that significantly positively correlated with pH and fungal growth (Table 1).

**Table 1:** Correlation analysis of CWDE activity with pH and mycelial dry weight in *R. solani* liquid shaken cultures with different carbon sources.

Carbon source	Correlation analysis					
			PG	PMG	Cx	β-glucosidase
Pectin	pH	*r*	0.86**	0.847**	0.34	0.873**
		*p*	0.003	0.004	0.371	0.002
	Mycelial dry weight	*r*	0.741*	0.855**	0.261	0.683*
		*p*	0.022	0.003	0.497	0.043
Pectin+CMC	pH	*r*	0.975**	0.973**	0.959**	0.662
		*p*	0.000	0.000	0.000	0.052
	Mycelial dry weight	*r*	0.963**	0.951**	0.937**	0.617
		*p*	0.000	0.000	0.000	0.077
CMC	pH	*r*	0.692*	0.881**	0.760*	0.640
		*p*	0.039	0.002	0.018	0.063
	Mycelial dry weight	*r*	0.674*	0.735*	0.856**	0.358
		*p*	0.047	0.024	0.003	0.344

**Notes:**

Liquid medium with various carbon sources including 1% pectin, 0.5% pectin + 0.5% CMC or 1% CMC were used in the experiment, grown at 25 °C with shaking at 100 rpm.

*r* = Correlation coefficient.

Correlation was analyzed using the Pearson correlation coefficient in SPSS 20.0 software.

* Correlation significant at the *p* < 0.05 level.

** Correlation significant at the *p* < 0.01 level.

### Effect of crude enzyme solution on tissue

**Figure 4:**
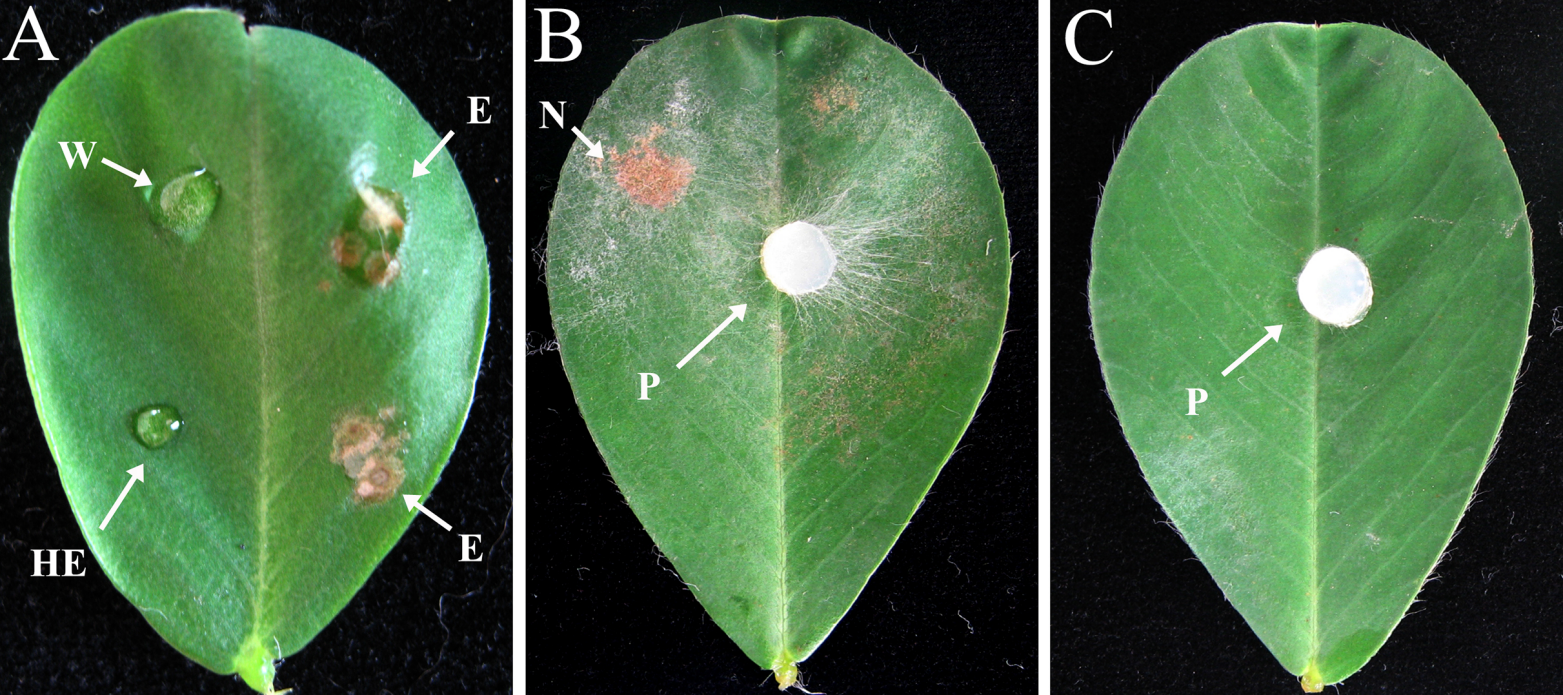
Effects of crude enzyme extract from *R. solani* culture and *R. solani* mycelium inoculated onto peanut leaf for 2 days. Leaves of Bs cultivar were treated with a five μl drop of *R. solani* crude enzyme extract or mycelial plugs five mm in diameter. The crude enzyme extracts were collected 10 dpi from medium containing pectin plus CMC as carbon sources incubated with *R. solani*. All treated leaves were incubated at 27 °C. (A) Crude enzyme extract inoculation. (B) Mycelial plug inoculation. (C) Sterile potato dextrose agar plug inoculation. W, distilled water (control); E, crude enzyme; HE, heated crude enzyme (control); N, necrotic lesion; P, mycelial plug. Treatment with “E” resulted in symptoms of necrotic lesions, leaves treated with “W” or “HE” showed no necrotic lesions.

Crude enzyme extract obtained from a 10 dpi culture containing pectin plus CMC was tested by inoculation onto peanut leaves. The crude enzymatic extract was able to induce symptoms of necrotic lesion, whereas the control samples such as heated enzyme and distilled water did not produce any symptoms of necrotic lesion (Fig. 4A), indicating the possible involvement of CWDEs in lesion development. The results also showed that the leaves treated with crude enzyme extract developed symptoms similar to those caused by infection with *R. solani* mycelium (Figs. 4A and 4B), while control leaves inoculated with sterile PDA plugs showed no lesions (Fig. 4C).

### Isoelectric focusing of polygalacturonase activity

**Figure 5:**
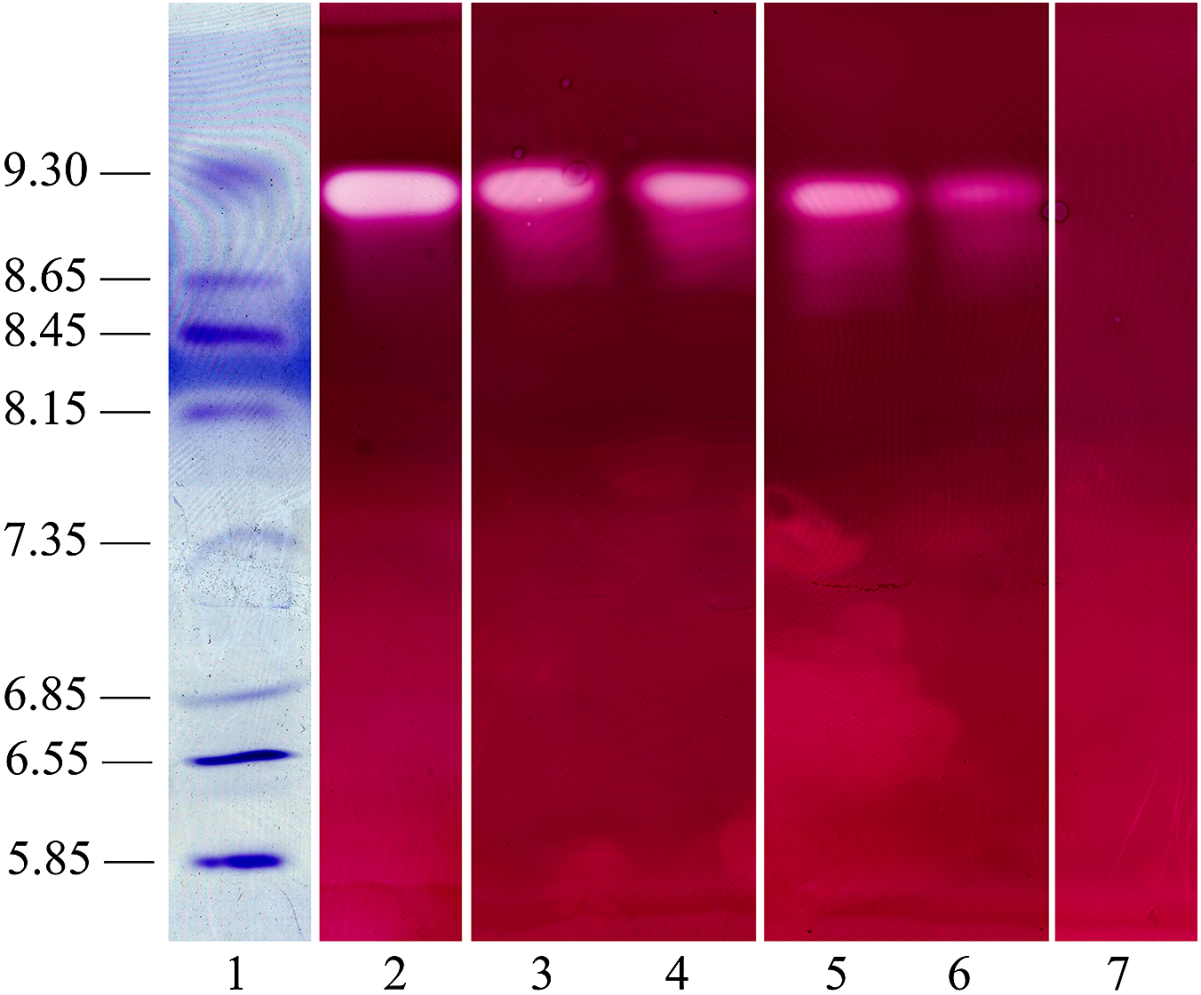
Polygalacturonase isozyme produced in shaken liquid cultures and in infected tissue inoculated with *R. solani* (both for 10 days). The crude enzyme extracts were collected from liquid medium containing pectin plus CMC as carbon sources incubated with *R. solani*. Samples were separated on an ultrathin layer polyacrylamide isoelectric focusing gel (pH 3.5–10.0), followed by agarose overlay activity staining. The PG band is white, visualized using a light box, with isoelectric point around 9.2. Lane 1, pI makers; 2, extract from culture filtrate; 3, extract from infected stalk of Baisha (Bs) cultivar; 4, extract from infected leaf of Bs cultivar; 5, extract from infected stalk of Silihong (Slh) cultivar; 6, extract from infected leaf of Slh cultivar; 7, control (heat-treated extract).

The dialyzed extracts collected 10 dpi from culture filtrates and from infected tissues including stems and leaves of Bs and Slh peanut cultivars were subjected to thin-layer polyacrylamide gel IEF and evaluated for the presence of PG isoenzymes. One PG band, with pI ∼9.2, was observed in all the samples (Fig. 5). *R. solani* produced the same single PG isoform in vitro (i.e., in shaken flask cultures) and during host–pathogen interaction. The control (heat-treated extracts) showed no PG band (Fig. 5). Experiments indicated that the same, single PG isoenzyme was induced in infected leaf tissue and in in vitro fungal cultivation.

## Discussion

Many previous studies have shown that *R. solani* as a pathogen isolated from many hosts produces CWDEs in vitro, in infected host tissue, or both (Brien & Zamani, 2003; Jayasinghe, Wijayaratne & Fernando, 2004; Balali & Kowsari, 2004; Chen et al., 2006; Zhao et al., 2014; Zhou et al., 2016). In this study, *R. solani* was determined to secrete a range of CWDEs including pectinase and Cx in both infected peanut tissue and culture medium.

*R. solani* produced high activities of PG and PMG in diseased peanut tissue, including leaves and stalks of two cultivars. Some pathogens have been determined to produce a high level of PG activity in culture medium, but activity could not be detected in any diseased host tissue because the host contained proteinaceous inhibitors of PG (Wijesundera et al., 1989). *C. acutatum*, *C. lindemuthianum* and *T. cucumeris* can produce high levels of PG activity in culture but not in infected host tissue, even though no proteinaceous inhibitors of PG were detected in the host (Fernando, Jayasinghe & Wijesundera, 2001; Jayasinghe, Wijayaratne & Fernando, 2004; Chilosi & Magro, 1998). It was suggested that PG had little function in these infections. However, in our study, high levels of both PG and PMG activity were observed in decayed peanut tissue, including leaf and stalk; thus, PG and PMG are suggested to play a significant role in the process of peanut sheath disease.

Compared to the pectolytic enzymes, we observed relatively weak Cx activities in infected tissue, especially β-glucosidase activity, which was also reported to be low in yam tissue infected by *Penicillium oxalicum* (Ikotun, 1984). Activities of PG, PMG and Cx were higher in stalk than in leaf in both cultivars investigated in the present work, whereas β-glucosidase activity was lower in stalk than in leaf. Moreover, we found in the present study that peanut leaf was more easily and more rapidly infected than stalk, possibly due to its structure (Qu & Zhang, 2012). Pectolytic enzymes are the first enzymes to be induced by fungal pathogens when they infect plant tissue as well as exposing cell wall components to other enzymes include Cx (Niture, Kumar & Pant, 2006; Jia et al., 2009). Thus, the activities of PG, PMG and Cx in leaf were lower than in stalk because of possible enzyme degradation induced by the different disease stages of peanut sheath blight. In addition, Cx degrades cellulose to cellobiose and then β-glucosidase degrades cellobiose into glucose monomers; thus, β-glucosidase expression occurs later than Cx expression during the progress of infection.

The presence of CWDEs has been reported in healthy tissue in several plants (Le Cam et al., 1997; Fernando, Jayasinghe & Wijesundera, 2001; Jayasinghe, Wijayaratne & Fernando, 2004; Lieberei et al., 1986; Amit et al., 2014). Consistent with these results, traces of CWDE activity were detected in healthy tissues in our study (although Cx could not be detected in the extract from leaves of the Bs cultivar), but we cannot exclude that this trace activity was due to the experimental conditions, for example, the use of a clear plastic bag to maintain high humidity for plant growth. Although plants activate the immune system when they encounter pathogenic microorganisms, including secreting enzyme (Shafikova & Omelichkina, 2015), in the current study the sampled tissues were necrotic and macerated due to infection by the pathogen. Therefore, we suggest that it is very likely that the major contribution to the overall quantity of the CWDEs present in the diseased tissue extract sis of fungal origin.

Our results indicate that *R. solani* can use pectin and CMC as carbon sources to produce a range of enzymes. We observed high PG and PMG activity when pectin was present in the medium, while high Cx activity was detected in culture containing CMC. In all three types of medium, β-glucosidase activity was hardly induced. Our results agreed with the reports of Dong et al. (2010) and Zhang, Bruton & Biles (2014). In contrast to the current results, Cx was not secreted by *Mycosphaerella graminicola* either in medium containing wheat cell walls or CMC as the carbon source (Douaiher et al., 2007a). From our data, we suggest that *R. solani* PG, PMG and Cx are substrate-inducible enzymes. However, in decayed tissue, we observed relatively high β-glucosidase activity. Therefore, β-glucosidase production by the fungus appears to require some other factors such as elicitors which are present in peanut tissues but not in laboratory culture.

The alkalization of culture and host tissue inoculated with fungal pathogens has been studied extensively (Wijesundera et al., 1989; Chilosi & Magro, 1998; Jurick et al., 2012; Zhang, Bruton & Biles, 2014). In the present study, we also observed a similar modulation pattern in the disease process. The pH increase may be attributed to the production of ammonia and amides by deamination reactions and metabolism respectively (Wijesundera et al., 1989; Prusky et al., 2001; Eshel et al., 2002), or to oligogalacturonide fragments from plant cell walls produced by pectolytic enzyme activity inducing rapid plant defensive reactions including extracellular alkalinization and an oxidative burst (Chilosi & Magro, 1998). Several reports determined that an increase in the host ambient pH is critical for fungal pathogenicity (Prusky et al., 2001; Eshel et al., 2002), and the varying pH may induce different types of pectolytic enzymes in infected tissues (Zhang, Bruton & Biles, 2014). In agreement with the previous reports, the pH increased by various degrees in all three cultures media tested in the current study. pH increase in culture medium during fungal growth may be due to differential uptake of cations and anions (Chilosi & Magro, 1998) and/or a decrease in polyuronide content and efflux of ammonia by the fungus (Jurick et al., 2012). In our study, the increase in pH is significantly correlated with the growth rate of the fungus in all three media. Therefore, it is likely that the amount of mycelium present was directly responsible for the quantity of uptake of cations and anions and efflux of ammonia, which affected the pH.

Fungi have evolved elegant mechanisms of regulating catabolic enzyme production using signaling pathways that are responsive to external cues (Jurick et al., 2012). External factors such as carbon, nitrogen and ambient pH have been shown to act as regulators of cell-wall-degrading enzymes secreted by fungal plant pathogens (Reymondcotton, Fraissinettachet & Fèvre, 1996; Vautard-Mey & Fèvre, 2003; Jurick et al., 2012). Previous findings showed that fungal growth and enzyme activity did not coincide and the enzymes could be induced by their substrate (Jurick et al., 2012; Wagner, Kusserow & Schäfer, 2000), while, culture pH affected fungal enzyme activity (Jurick et al., 2012). We suggest that substrate is the predominant inducer of PG, PMG and Cx production by *R. solani*, because when the relevant substrate was present in the culture medium, significant enzyme activity was detected. In addition to this, either the pH or fungal growth or both affected the production of enzymes, especially PG, PMG and Cx activity. The regulatory effects of external cues on CWDE production by *R. solani* require further study.

Crude enzyme extracts from medium inoculated with fungal pathogens could damage the cell structure of plant tissue or cause disease symptoms (Chen et al., 2006; Jia et al., 2009). The present study provided the first evidence that CWDEs are produced by *R. solani* in peanut sheath blight and that they are likely responsible for the death and maceration of infected tissue. The crude enzymes include a series of pectolytic and cellulolytic enzymes but may also contain other toxins. However, reported *Rhizoctonia*-toxins were low-molecular-weight, thermally stable and nonprotein (Vidhyasekaran et al., 1997; Liang & Zheng, 2012; Liang et al., 2015; Kankam et al., 2016), while the crude enzymes used for inoculation in our experiments contained biomacromolecules prepared using a 10,000 Da MWCO and the crude enzyme control extract was heated. Thus, we suspect that CWDEs are important factors for sheath blight disease caused by the *R. solani* isolate. It will be beneficial to further explore the pathogenic mechanisms of *R. solani* during infection of peanuts.

Polygalacturonase is a principal enzyme of many fungal pathogens of plants. Previous studies have determined the important role of the PGs by molecular methods (Shieh et al., 1997; Di Pietro & Roncero, 1998; Oeser et al., 2002; Yang et al., 2012a; Chen et al., 2014, 2017a, 2017b). Extensive studies have been carried out into the types of PG isozymes in vivo and in vitro. Most fungal pathogens of plants were demonstrated to be capable of secreting multiple PG isozymes with different pI values, while some produced a single isozyme in culture (Jurick et al., 2012; Martel, Létoublon & Fěvre, 1996; Schnitzhofer et al., 2007; Cabanne & Donèche, 2002). Some pathogens were reported to produce different PG isozymes in different cultures depending on the environmental factors (Zhang et al., 1999; Chilosi & Magro, 1998; Jurick et al., 2010, 2012). Moreover, some pathogens produced different PG isozymes in vivo and in vitro, and in different hosts, due to environmental factors and elicitors (Dong et al., 2010; Jurick et al., 2010). It is reported that *R. solani* isolates produced multiple PG isoenzymes in vivo or in vitro (Scala et al., 1980; Balali & Kowsari, 2004). In our study, *R. solani* produced a single detectable isozyme in culture and in infected tissue, with an apparent pI of ∼9.2, which suggested that the same PG isoenzyme was induced in all the experimental conditions described. We suggest that the PG isoenzyme in the current study is very likely to play a significant role in infection. This is the first report on the PG isoenzyme produced by *R. solani* in peanut sheath disease, and it will provide an important basis for further purification and functional identification of PG in the host.

To our knowledge, this is the first investigation on the production of CWDEs by *R. solani* causing peanut sheath disease in vivo and in vitro. This study provides the basis for further study of the biochemical properties of the PG and its possible function in development of peanut sheath blight disease.

## Conclusion

In this study, we demonstrate that *R. solani*, the pathogen of peanut sheath blight, secretes a range of CWDEs including pectinase and Cx in vivo and in vitro. *R. solani* can produce significant PG, PMG, Cx and β-glucosidase activities during the development of peanut sheath blight disease. In shaken liquid cultures, substrate is the predominant inducer of PG, PMG and Cx production by *R. solani*, while only a very low level of β-glucosidase activity was observed in cultures with any of the tested carbon sources. An increase of pH was recorded in decayed peanut tissues and liquid culture filtrates; the filtrate pH and fungal growth positively correlated. The fungal growth and/or pH were important factors for the production of PG, PMG and Cx in culture with pectin plus CMC as carbon sources. A single active PG isozyme with isoelectric point around 9.2 was detected in culture filtrates and in infected peanut tissues by the method of IEF electrophoresis, which suggests that the PG isoenzyme found in the current study is very likely to play a significant role in infection. Crude enzymes containing pectinase and Cx extracted from liquid culture of *R. solani* induced decay of healthy peanut leaves and these enzymes are very likely responsible for the death and maceration of infected tissue in peanut sheath blight disease.

## Supplemental Information

10.7717/peerj.5580/supp-1 Supplemental Information 1 The calculation method and results of target protein pI.

10.7717/peerj.5580/supp-2 Supplemental Information 2 IEF gel contains pI maker and agarose overlay contains target protein.

10.7717/peerj.5580/supp-3 Supplemental Information 3 The agarose overlay stained by ruthenium red solution (contain control treatment).

10.7717/peerj.5580/supp-4 Supplemental Information 4 Effects of crude enzyme extract from *R. solani* culture inoculated onto peanut leaf for 2 days.

10.7717/peerj.5580/supp-5 Supplemental Information 5 Effects of *R. solani* mycelia plug inoculated onto peanut leaf for 2 days.

10.7717/peerj.5580/supp-6 Supplemental Information 6 Effects of sterile PDA inoculated onto peanut leaf for 2 days.

10.7717/peerj.5580/supp-7 Supplemental Information 7 18S rRNA, internal transcribed spacer 1, 5.8S rRNA, internal transcribed spacer 2, 28S rRNA, partial and complete sequence of Rhizoctonia solani, the pathogen of peanut sheath blight disease.

## Additional Information and Declarations

### Data Availability

The following information was supplied regarding data availability: The raw data are provided in the Supplemental Files.
