# Supplementary figures and images for: Cell-wall-degrading enzymes produced in vitro and in vivo by Rhizoctonia solani, the causative fungus of peanut sheath blight
Source: PeerJ. 2018 Sep 5;6:e5580. doi: 10.7717/peerj.5580 (PMC6129149; doi:10.7717/peerj.5580)

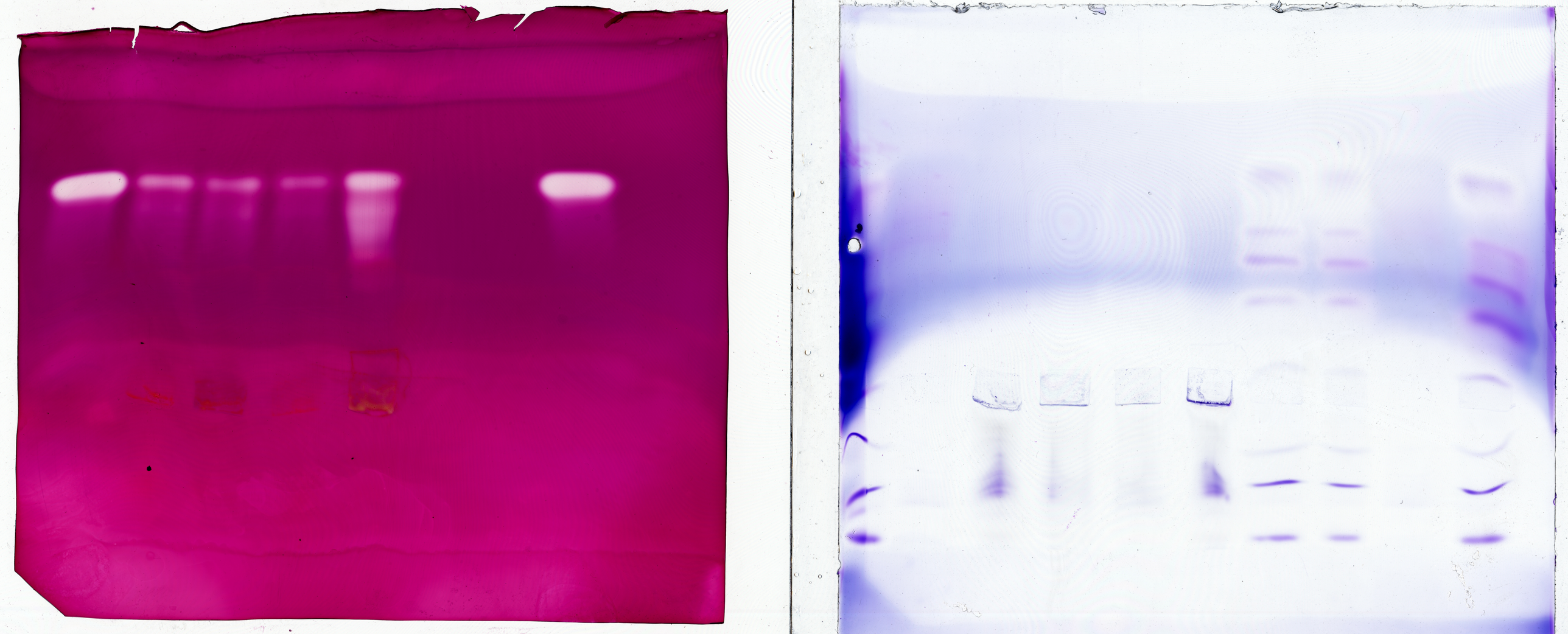

Supplement: Supplemental Information 2 [file peerj-06-5580-s002.jpg]

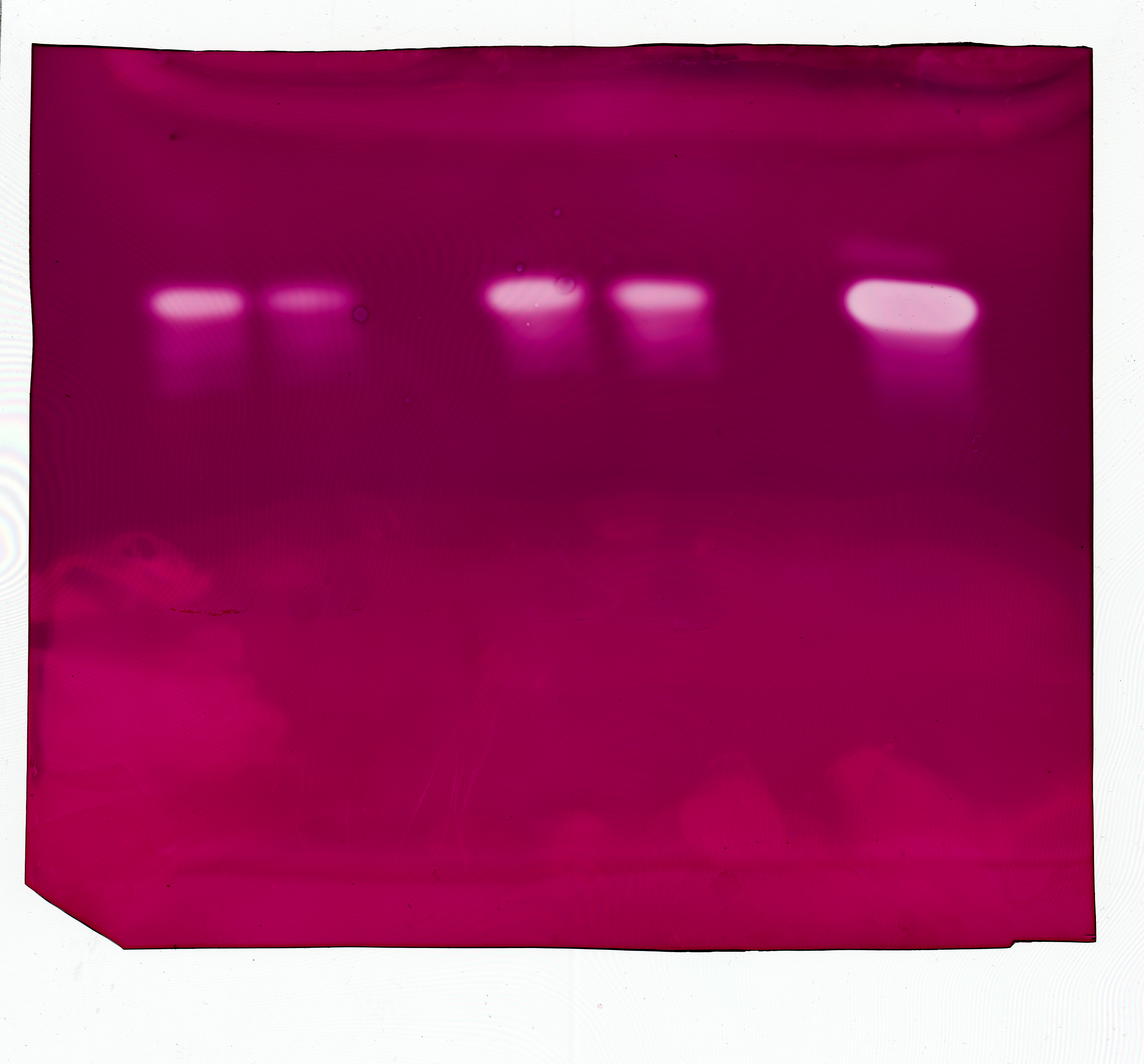

Supplement: Supplemental Information 3 [file peerj-06-5580-s003.jpg]

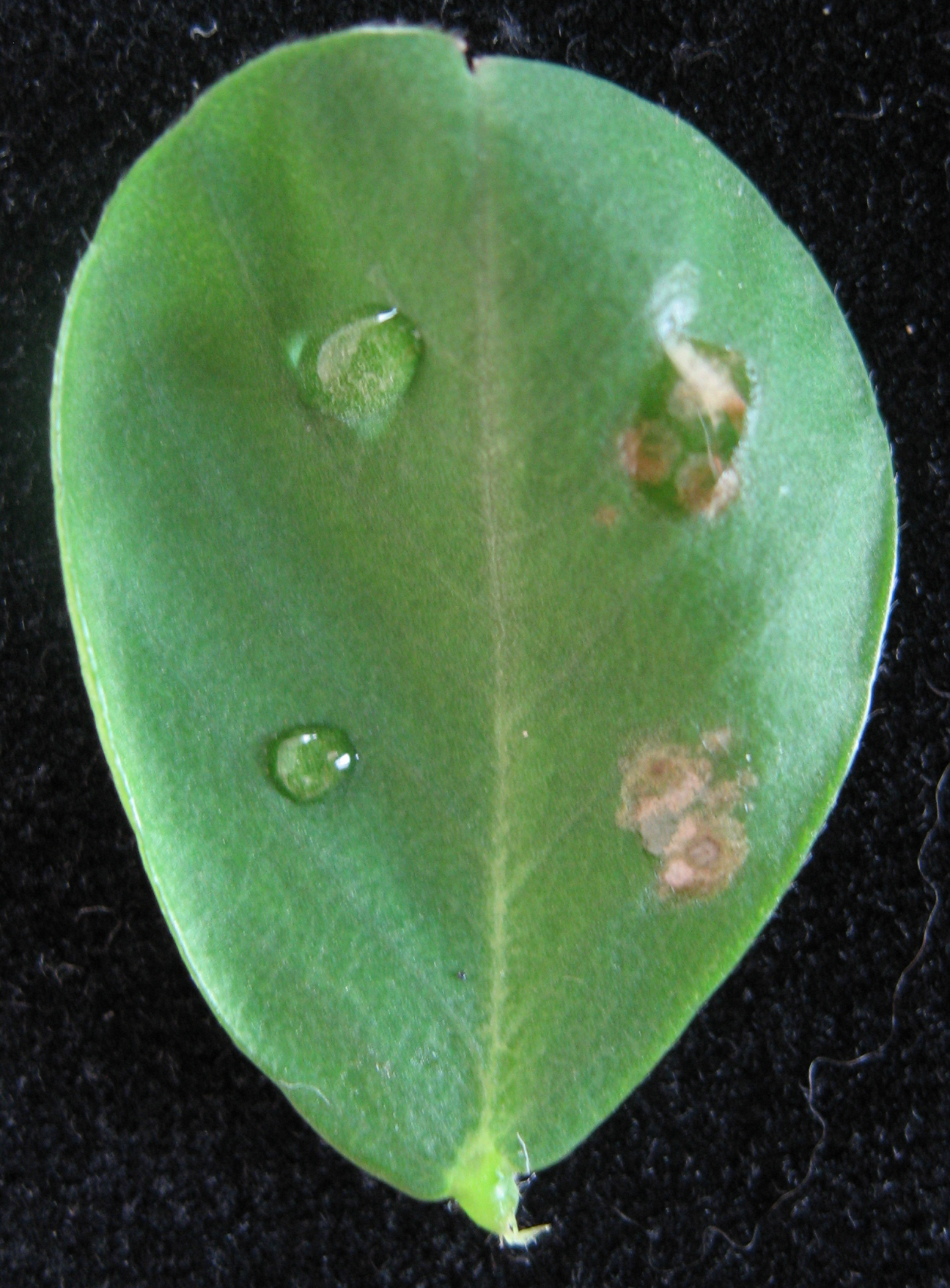

Supplement: Supplemental Information 4 [file peerj-06-5580-s004.jpg]

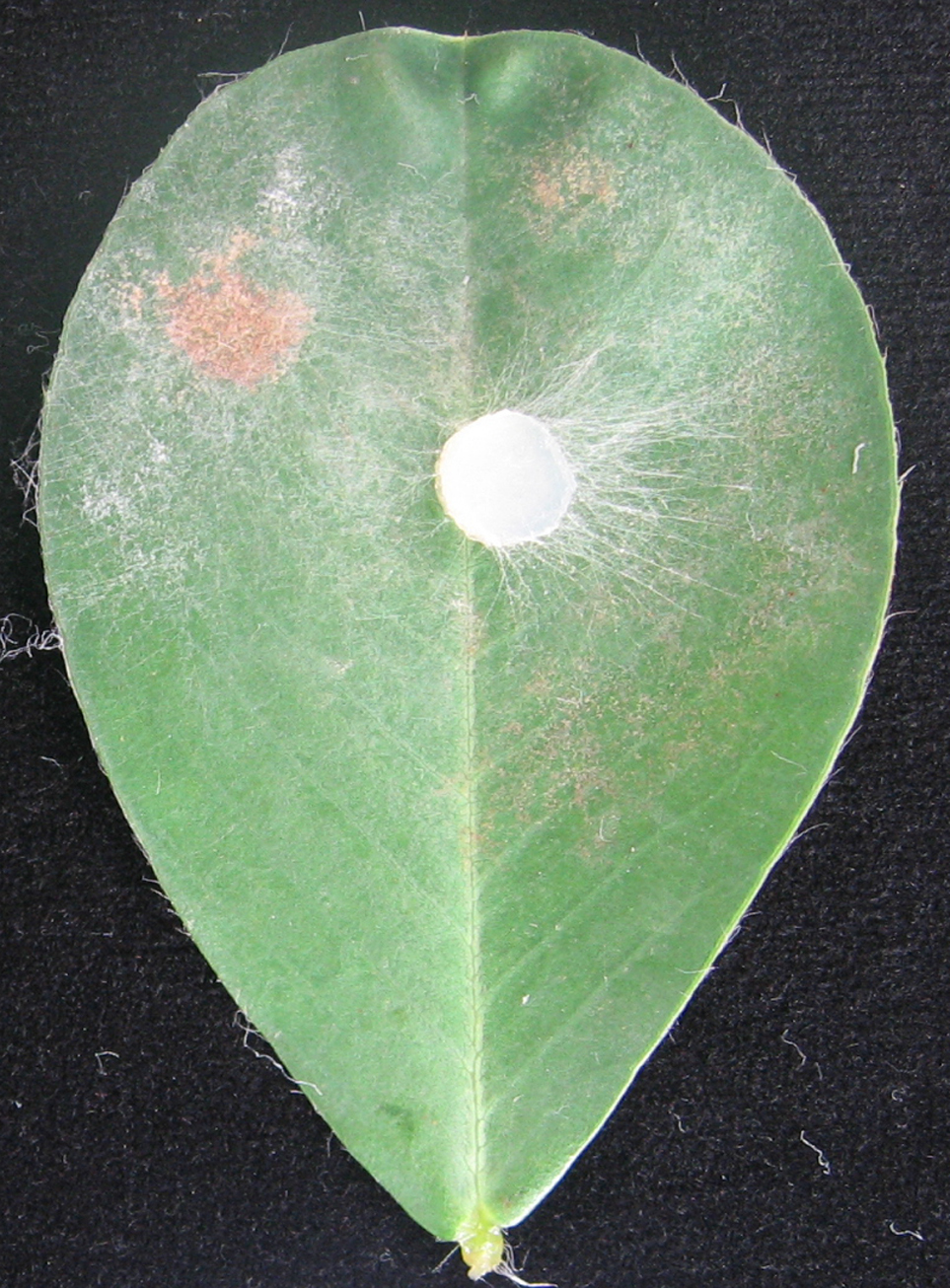

Supplement: Supplemental Information 5 [file peerj-06-5580-s005.jpg]

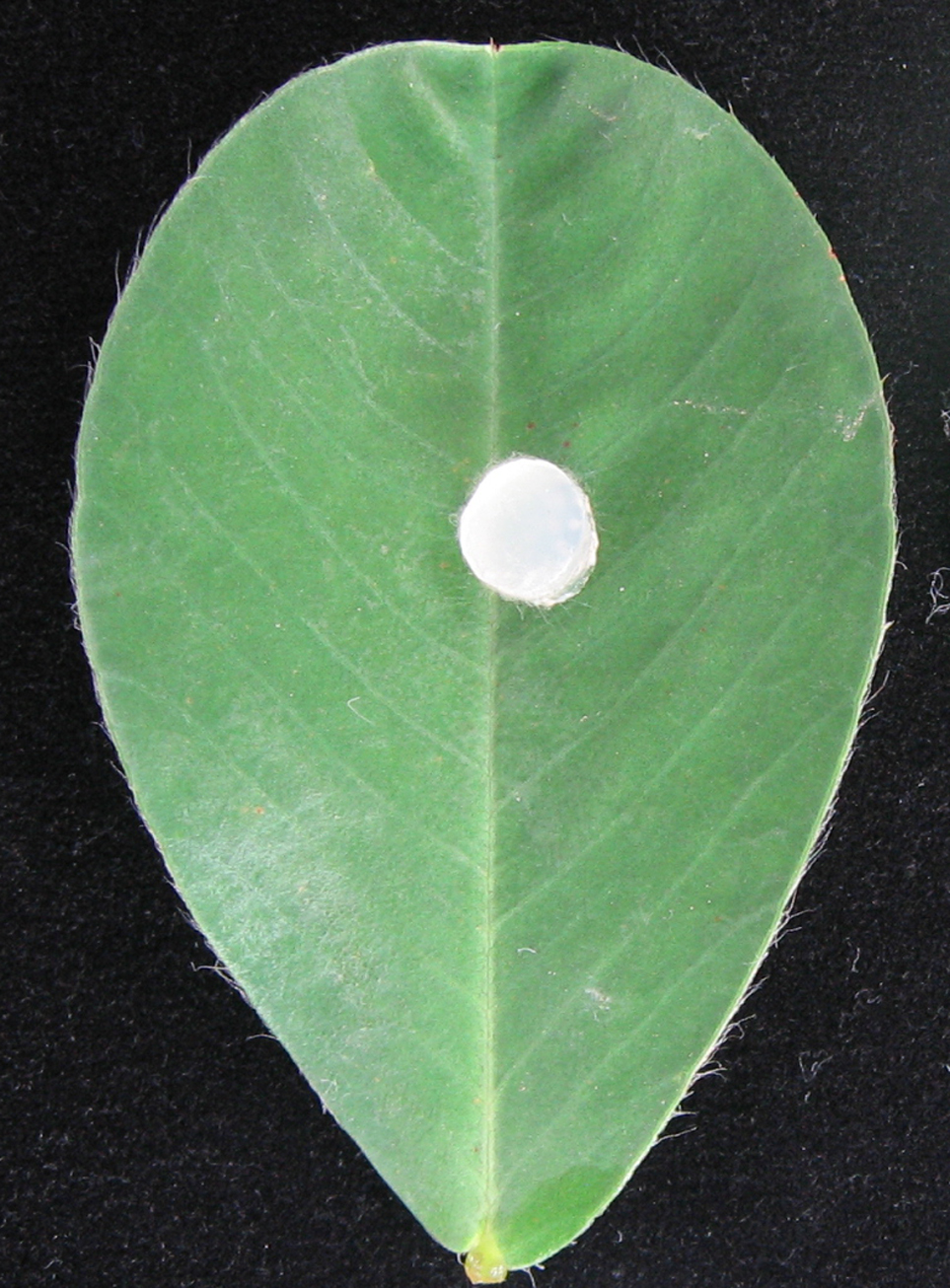

Supplement: Supplemental Information 6 [file peerj-06-5580-s006.jpg]
