# Supplementary material for: Cell-wall-degrading enzymes produced in vitro and in vivo by Rhizoctonia solani, the causative fungus of peanut sheath blight
Source: PeerJ. 2018 Sep 5;6:e5580. doi: 10.7717/peerj.5580 (PMC6129149; doi:10.7717/peerj.5580)
Supplement: Supplemental Information 7 [file peerj-06-5580-s007.docx]

TTCCGTAGGTGAACCTGCGGAAGGATCATTATTGAATTTTATTAATGAGGAGTTGAGTTGTTGCTGGCCTTTTCTACCTTAACTTGGCAGGAGGGGCATGTGCACACCTTCTCTTTCATCCATCACACCCCCTGTGCACTT

GTGAGACAGCAATAGTTGGTGGATTTAATTCCATCATCCGTTTGCTGTCTACTTAATTTACACACACTCTACTTAATTTAAACTGAATGTAATTGATGTAACGCATCTAATACTAAGTTTCAACAACGGATCTCTTGGCTC

TCGCATCGATGAAGAACGCAGCGAAATGCGATAAGTAATGTGAATTGCAGAATTCAGTGAATCATCGAATCTTTGAACGCACCTTGCGCTCCTTGGTATTCCTTGGAGCATGCCTGTTTGAGTATCATGAAATCTTCAAAG

TAAACCTTTTGTTAATTCAATTGGTCTTTTTTACTTTGGTTTTGGAGGATCTTATTGCAGCTTCACACCTGCTCCTCTTTGTGCATTAGCTGGATCTCAGTGTTATGCTTGGTTCCACTCGGCGTGATAAGTTATCTATCG

CTGAGGACACCCGTAAAAAAGGTGGCCAAGGTAAATGCAGATGAACCGCTTCTAATAGTCCATCGACTTGGACAATATTCTATTTTATGATCTGATCTCAAATCAGGTAGGACTACCCGCTGAACTTAAGCATATCAATAA

GCGGAGGAA
